# Alanine aminotransferase, HCV RNA levels and pro-inflammatory and pro-fibrogenic cytokines/chemokines during acute hepatitis C virus infection

**DOI:** 10.1186/s12985-016-0482-x

**Published:** 2016-02-24

**Authors:** Behzad Hajarizadeh, François MJ Lamoury, Jordan J. Feld, Janaki Amin, Elizabeth Keoshkerian, Gail V. Matthews, Margaret Hellard, Gregory J. Dore, Andrew R. Lloyd, Jason Grebely, Tanya L. Applegate, on behalf of the ATAHC Study Group

**Affiliations:** The Kirby Institute, UNSW Australia, Wallace Wurth Building, Sydney, 2052 Australia; Toronto Centre for Liver Disease, McLaughlin-Rotman Centre for Global Health, University of Toronto, Toronto, Canada; Inflammation and Infection Research Centre, School of Medical Sciences, UNSW Australia, Sydney, Australia; HIV/Immunology/Infectious Diseases Clinical Services Unit, St Vincent’s Hospital, Sydney, Australia; Burnet Institute, Melbourne, Australia

**Keywords:** HCV, ALT, Interferon-gamma inducible protein-10, IP-10, Macrophage inflammatory protein 1beta, MIP-1β

## Abstract

**Background:**

This study assessed the association of alanine-aminotransferase (ALT) and hepatitis C virus (HCV) RNA levels with pro-inflammatory and pro-fibrogenic cytokines and chemokines during acute HCV infection to provide further insight into the potential HCV immunopathogenesis.

**Methods:**

Participants in the ATAHC study, a prospective study of recent HCV infection, with detectable HCV RNA at the time of HCV detection were included. Plasma levels of 27 cytokines and chemokines were measured and their correlation with ALT and HCV RNA levels were assessed. Log_10_ transformed cytokines and ALT values were used in the analysis.

**Results:**

Among 117 individuals, the plasma levels of interferon-gamma inducible protein-10 (IP-10) and macrophage inflammatory protein-1beta (MIP-1β) were positively correlated with ALT levels (IP-10: *r* = 0.42, *P* < 0.001; MIP-1β: *r* = 0.29, *P* = 0.001) and HCV RNA levels (IP-10: *rs* = 0.44, *P* < 0.001; MIP-1β: *rs* = 0.43, *P* < 0.001). Using linear regression, after adjusting for sex, age, infection duration, symptomatic infection, HIV co-infection, *interferon*-*lambda rs12979860* genotype, HCV genotype, and assay run, higher ALT levels (β = 0.20; 95 % CI: 0.07, 0.32; *P* = 0.002) and HCV RNA levels >400,000 IU/mL (vs. <8,500 IU/mL; β = 0.16; 95 % CI: 0.03, 0.28; *P* = 0.014) were independently associated with higher IP-10 levels. HCV RNA levels >400,000 IU/mL (vs. <8,500 IU/mL; β = 0.16; 95 % CI: 0.01, 0.31; *P* = 0.036) were associated with higher MIP-1β levels.

**Conclusions:**

During acute HCV infection, high ALT and HCV RNA levels were associated with increased IP-10 levels, while high HCV RNA levels were also associated with increased MIP-1β levels. These data suggest that IP-10 and MIP-1β may have a role in HCV immuno-pathogenesis starting early in acute HCV infection.

**Electronic supplementary material:**

The online version of this article (doi:10.1186/s12985-016-0482-x) contains supplementary material, which is available to authorized users.

## Background

The majority of individuals with acute hepatitis C virus (HCV) infection progress to chronic infection [1, 2], and are at increased risk of liver fibrosis progression [3, 4]. Several studies have demonstrated that while elevation of alanine aminotransferase (ALT) is an important predictor of the development and progression of liver fibrosis in chronic HCV infection, HCV RNA levels have limited role in this regard (reviewed in [4, 5]). This supports a hypothesis suggesting a more important role of intra-hepatic inflammation in development and progression of liver fibrosis than direct HCV cytotoxicity in HCV infection.

In a longitudinal study investigating six individuals with acute HCV infection followed from HCV acquisition up to 30 years following infection, rapid liver disease progression correlated with persistent elevation of ALT levels [6]. These data suggest that a persistently high ALT during acute HCV infection is a marker for enhanced liver disease progression in the later stages of chronic HCV infection. Further investigation of the factors associated with ALT levels in acute HCV infection may provide a better understanding of immuno-pathogenesis of HCV infection.

The underling mechanisms promoting the development of hepatic inflammation in HCV infection have not been well understood. Cytokines and chemokines may have a role in the development of HCV-induced liver inflammation (reflected by ALT elevation) given their role in recruitment, accumulation, and localisation of inflammatory cells in the liver following HCV infection (reviewed in [7, 8]). In the setting of chronic HCV infection, several studies identified a correlation between ALT and/or HCV RNA levels with peripheral concentrations of several cytokines [8–16] while the up-regulation of intra-hepatic expression of a number of cytokines have been reported in patients with HCV-induced advanced liver inflammation and fibrosis [17–19]. However, in the setting of acute HCV infection, there are relatively little data on the role of cytokines and chemokines in liver inflammation and the association of cytokines and chemokines with ALT or HCV RNA levels, while the existing studies have been limited by small sample sizes [6, 20–23].

The present study was undertaken to assess the association of ALT and HCV RNA levels with plasma levels of pro-inflammatory and pro-fibrogenic cytokines and chemokines at the time of HCV detection in recent HCV infection (i.e., acute and early chronic).

## Results

### Participant characteristics

A total of 117 individuals with recent HCV infection were included in the study. Background characteristics of the study population are summarized in Table 1. In brief, the median age was 34 years, 75 % were male, 42 % had symptomatic illness at HCV detection, 38 % had HIV co-infection, and 57 % were infected with HCV genotype 1. Median ALT and HCV RNA levels at the time of HCV detection were 145 IU/L [inter-quartile range (IQR): 73 – 385] and 73,760 IU/ml (IQR: 3,269 - 805,345), respectively. Seventy-five percent (n = 33) of individuals with HIV co-infection were on antiretroviral therapy at the time of HCV detection.

**Table 1:** Baseline characteristics of ATAHC participants with detectable HCV RNA at the time of acute HCV detection

	Number (%) *Total n* = *117*
Sex	
Male	88 (75)
Female	29 (25)
Median age, yrs (IQR)	34 (26–42)
Symptomatic acute HCV	
No	68 (58)
Yes	49 (42)
Estimated duration of infection at acute HCV detection	
<26 weeks	69 (59)
≥26 weeks	48 (41)
HIV co-infection	
Negative	73 (62)
Positive	44 (38)
*Interferon lambda rs12979860* genotype	
TT/CT	56 (48)
CC	59 (50)
Unknown	2 (2)
Median ALT level at acute HCV detection, IU/L (IQR)	145 (73–385)
Median HCV RNA level at acute HCV detection, IU/mL (IQR)	73,760 (3,269-805,345)
HCV genotype	
Genotype 1	67 (57)
Genotype 2	6 (5)
Genotype 3	40 (34)
Other genotypes^a^	2 (2)
Unknown genotype	2 (2)

Abbreviation: *IQR* inter-quartile range, ^a^Including genotype 4 (n = 1) and mixed genotype (n = 1)

### Factors associated with ALT and HCV RNA levels

Factors associated with ALT levels (Additional file 1: Table S2) and HCV RNA levels (categorised by tertile, Additional file 1: Table S3) were assessed. Male gender, older age, symptomatic HCV, HIV co-infection, estimated duration of infection <26 weeks, and higher HCV RNA levels were significantly associated with higher ALT levels (Additional file 1: Table S2).

Distributions of gender, age and ALT levels were significantly different across HCV RNA tertiles while male gender, older age, HIV co-infection, and higher ALT levels were significantly associated with HCV RNA levels >400,000 IU/mL (Additional file 1: Table S3).

### Correlation of ALT and HCV RNA levels with cytokines and chemokines

**Fig. 1:**
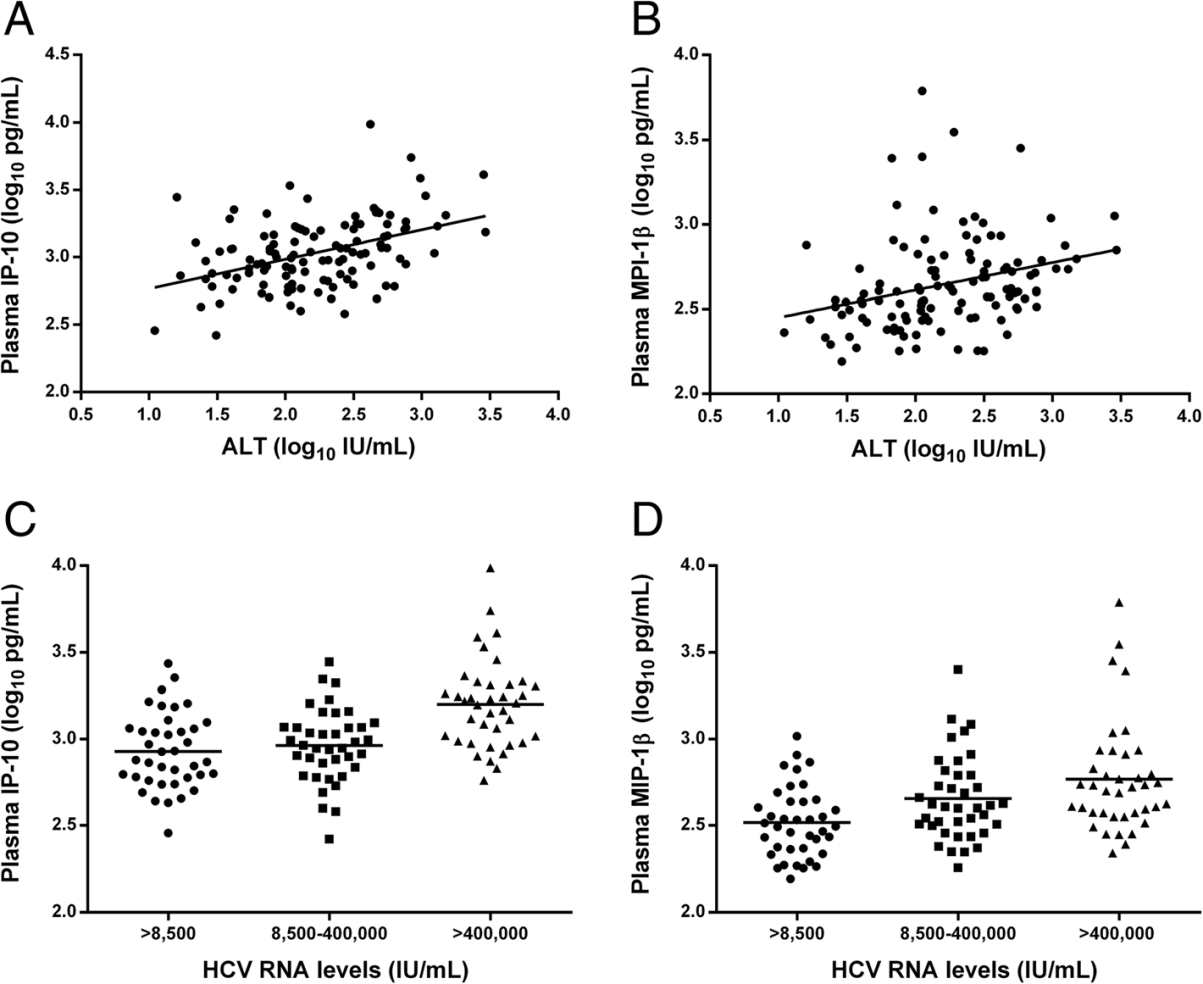
Association of ALT and HCV RNA levels with IP-10 and MIP-1β levels in ATAHC participants with detectable HCV RNA at the time of acute HCV detection (n = 117). (**a**) Distribution of IP-10 levels by ALT levels (Pearson’s *r* = 0.43, *P* < 0.001); (**b**) Distribution of MIP-1β levels by ALT levels (Pearson’s *r* = 0.29, *P* = 0.001); (**c**) Distribution of IP-10 levels by HCV RNA tertiles (*P between groups* < 0.001); (**d**) Distribution of MIP-1β levels by HCV RNA tertiles (*P between groups* < 0.001). Oblique lines in A and D represent the best fit linear line. Horizontal lines in C and D represent the means cytokines levels in each subgroup

Correlation of ALT and HCV RNA levels with plasma cytokine and chemokine levels is summarized in Table 2. ALT levels were positively correlated with IP-10 levels (Pearson’s *r* = 0.43, *P* < 0.001; Fig. 1a) and MIP-1β levels (Pearson’s *r* = 0.29, *P* = 0.001; Fig. 1b). Correlation of ALT levels with IP-10 levels was stronger than that with MIP-1β levels. HCV RNA levels were also positively correlated with IP-10 levels (Spearman’s *rho* = 0.44, *P* < 0.001) and MIP-1β levels (Spearman’s *rho* =0.43, *P* = 0.001). Mean IP-10 levels (*P* < 0.001; Fig. 1c) and mean MIP-1β levels (*P* < 0.001, Fig. 1d) were significantly different between HCV RNA tertiles with increasing trends in means being observed from the lowest tertile to the top tertile.

**Table 2:** Correlation of ALT and HCV RNA levels with cytokine and chemokine concentrations in ATAHC participants with detectable HCV RNA at the time of acute HCV detection (n = 117)

Cytokine	ALT		HCV RNA	
	Pearson’s correlation coefficient^a^	*P*	Spearman’s rho^b^	*P*
IL-1β	−0.05	0.594	−0.02	0.800
IL-2	−0.16	0.089	0.03	0.755
IL-4	0.07	0.459	−0.03	0.761
IL-6	−0.07	0.436	0.13	0.179
IL-8	0.08	0.400	0.20	0.029
IL-10	0.04	0.668	−0.07	0.460
IL-17A	−0.05	0.609	0.00	0.998
IL-17 F	−0.13	0.180	0.08	0.388
IL-18	0.02	0.848	−0.06	0.540
IL-21	0.05	0.629	0.01	0.943
IL-22	0.03	0.735	0.08	0.400
IL-25	0.01	0.921	0.05	0.629
IL-31	−0.12	0.198	−0.06	0.541
IL-33	0.00	0.982	0.00	0.973
IFN-γ	−0.01	0.904	−0.09	0.359
TNF-α	−0.03	0.780	0.11	0.240
TRAIL	−0.01	0.912	−0.15	0.118
sCD40L	0.04	0.680	0.06	0.539
Eotaxin (CCL11)	0.06	0.534	0.05	0.574
IP-10 (CXCL10)	0.43	<0.001	0.44	<0.001
MCP1 (CCL2)	0.18	0.049	0.13	0.173
MIP-1β (CCL4)	0.29	0.001	0.43	<0.001
RANTES (CCL5)	0.11	0.232	0.01	0.897
	Median (IQR)^c^	*P*	Median (IQR)^d^	*P*
IL-23		0.066		0.860
BLL (n = 30)	278 (109–498)		84,615 (6,538-692,308)	
Quantified (n = 86)	130 (67–325)		74,380 (2,218-826,923)	
IFN-γ2		0.315		0.277
BLL (n = 95)	153 (82–355)		67,308 (2,785-634,615)	
Quantified (n = 21)	130 (54–385)		364,433 (8,654-1,288,462)	
TNF-β		0.384		0.174
BLL (n = 111)	145 (73–423)		88,756 (2,885-826,923)	
Quantified (n = 6)	125 (70–254)		11,442 (5,836-14,423)	
MIP-1α (CCL3)		0.048		0.170
BLL (n = 52)	115 (54–296)		41,752 (2,732-395,529)	
Quantified (n = 65)	217 (77–486)		88,756 (3,519-1,057,692)	

Abbreviation: *IQR* inter-quartile range, *BLL* below lower limit of quantification

^a^ *ALT* log_10_ IU/L, *Cytokines* log_10_ pg/mL, ^b^ *HCV RNA* IU/mL, *Cytokines* pg/mL, ^c^ *ALT* IU/L, ^d^ *HCV RNA* IU/mL

IP-10 and MIP-1β were selected for further analysis. In unadjusted linear regression analyses using IP-10 levels as the outcome variable, higher ALT levels (β = 0.21; 95 % CI: 0.13, 0.30; *P* < 0.001) and HCV RNA >400,000 IU/mL (vs. < 8,500 IU/mL; β = 0.27; 95 % CI: 0.17, 0.37; *P* < 0.001) were significantly associated with higher IP-10 levels (Table 3). In a model adjusting for sex, age, estimated duration of infection, symptomatic infection, HIV co-infection, *interferon*-*lambda rs12979860* genotype, HCV genotype, and assay run, higher ALT levels (adjusted β = 0.20; 95 % CI: 0.07, 0.32; *P* = 0.002) and HCV RNA >400,000 IU/mL (vs. < 8,500 IU/mL; adjusted β = 0.16; 95 % CI: 0.03, 0.28; *P* = 0.014) remained independently associated with higher IP-10 levels (Table 3).

**Table 3:** Unadjusted and adjusted models assessing the correlation of plasma IP-10 and MIP-1β levels (log pg/mL) with ALT and HCV RNA levels in ATAHC participants with detectable HCV RNA at the time of acute HCV detection

	Unadjusted model		Adjusted model^*^	
	Estimated Mean difference^**^ (95 % CI)	*P*	Estimated Mean difference^**^ (95 % CI)	*P*
IP-10 level, log pg/mL				
ALT level, log IU/L	0.21 (0.13, 0.30)	<0.001	0.20 (0.07, 0.32)	0.002
HCV RNA level				
<8,500 IU/mL	Reference		Reference	
8,500 - 400,000 IU/mL	0.03 (−0.07, 0.14)	0.512	−0.05 (−0.17, 0.07)	0.414
>400,000 IU/mL	0.27 (0.17, 0.37)	<0.001	0.16 (0.03, 0.28)	0.014
MIP-1β level, log pg/mL				
ALT level, log IU/L	0.16 (0.06, 0.26)	0.001	0.09 (−0.06, 0.24)	0.224
HCV RNA level				
<8,500 IU/mL	Reference		Reference	
8,500 - 400,000 IU/mL	0.14 (0.02, 0.26)	0.020	0.10 (−0.04, 0.24)	0.165
>400,000 IU/mL	0.25 (0.13, 0.37)	<0.001	0.16 (0.01, 0.31)	0.036

*Adjusted for variables associated with the outcome cytokine (i.e., IP-10 or MIP-1β), ALT or HCV RNA levels (*P* < 0.200) in unadjusted analysis, including sex, age, symptomatic acute HCV, estimated duration of infection, HIV co-infection, *Interferon lambda rs12979860* genotype, HCV genotype, and assay run; n = 113; R^2^ = 0.40 (for the IP-10 model); R^2^ = 0.28 (for the MIP-1β model)

**β coefficient

Using MIP-1β levels as the outcome variable in unadjusted linear regression analyses, higher ALT levels (β = 0.16; 95 % CI: 0.06, 0.26; *P* = 0.001) and higher HCV RNA levels were significantly associated with higher MIP-1β levels, while mean MIP-1β levels significantly increased by HCV RNA tertiles (8,500-400,000 vs. < 8,500 IU/mL: β = 0.14; 95 % CI: 0.02, 0.26; *P* = 0.020; >400,000 vs. < 8,500 IU/mL: β = 0.25; 95 % CI: 0.13, 0.36; *P* < 0.001; Table 3). In a model adjusting for sex, age, estimated duration of infection, symptomatic infection, HIV co-infection, *interferon*-*lambda rs12979860* genotype, HCV genotype, and assay run, only HCV RNA >400,000 IU/mL (vs. < 8,500 IU/mL; adjusted β = 0.16; 95 % CI: 0.01, 0.31; *P* = 0.036) remained significantly associated with higher MIP-1β levels. Mean MIP-1β levels was not significantly different between HCV RNA levels 8,500-400,000 IU/mL vs. < 8,500 IU/mL although an increasing trend in mean MIP-1β levels by HCV RNA tertiles was observed (Table 3).

In a sensitivity analysis, the correlations of IP-10 and MIP-1β with ALT and HCV RNA levels were assessed, stratified by HIV co-infection status (Additional file 1: Table S4). In both groups of HCV mono-infection and HCV/HIV co-infection, higher ALT levels and HCV RNA >400,000 IU/mL were significantly associated with higher IP-10 levels. The correlation of ALT with IP-10 levels was relatively stronger in HCV/HIV co-infection group (β = 0.26; 95 % CI: 0.10, 0.41) compared to that in HCV mono-infection group (β = 0.17; 95 % CI: 0.06, 0.28).

Higher ALT and HCV RNA levels were significantly associated with higher MIP-1β levels in HCV mono-infection group. In HCV/HIV co-infection, only HCV RNA >400,000 IU/mL was significantly associated with higher MIP-1β levels.

In another sensitivity analysis, all cytokines and chemokines were included in a linear regression model with ALT being the outcome variable. In adjusted analysis, IP-10 and MIP-1β were the only cytokines significantly associated with ALT (*P* < 0.001 and *P* = 0.002, respectively). A logistic regression model was also developed with HCV RNA (≤400,000 IU/mL vs. >400,000 IU/mL) as the outcome variable and all cytokines and chemokines as the study variables. In adjusted analysis, higher IP-10 and MIP-1β were significantly associated with HCV RNA > 400,000 IU/mL (*P* < 0.001 and *P* = 0.004, respectively).

## Discussion

This study assessed the association of ALT and HCV RNA levels with plasma levels of several pro-inflammatory and pro-fibrogenic cytokines and chemokines in recent HCV infection. Higher ALT levels were independently associated with higher IP-10 levels while high HCV RNA levels (i.e., >400,000 IU/mL) were independently associated with higher IP-10 and MIP-1β levels. These findings suggest that the potential role of IP-10 and MIP-1β in HCV immuno-pathogenesis starts from acute phase of infection, improving our understanding of HCV immuno-pathogenesis.

This study identified independent associations between higher ALT and HCV RNA levels with higher IP-10 levels in acute HCV infection. IP-10 (CXCL10) and its unique receptor CXCR3 have important roles in recruitment of monocytes, macrophages, T lymphocytes, natural killer cells, and dendritic cells to the inflamed tissue (reviewed in [24]). The role of IP-10 in the recruitment of activated T cells is particularly important in immuno-pathogenesis of HCV infection given the central role of innate and adaptive immune response in controlling HCV replication in acute infection and in progression of liver disease in chronic infection [8, 25, 26]. The role of IP-10 in immuno-pathogenesis of HCV infection has been well-defined given the evidence demonstrating the elevation of plasma IP-10 levels after HCV acquisition, potentially linking IP-10 elevation to innate immunity [20–22], and the association of plasma levels and/or intrahepatic expression of IP-10 with spontaneous HCV clearance in acute infection [27, 28] and with interferon-induced viral control in chronic infection [13, 29, 30]. Further, IP-10 is suggested to have a role in development of hepatic necro-inflammation in chronic HCV infection given that several studies demonstrated an association between increased plasma IP-10 levels [9–11, 13, 29, 31] and upregulated intra-hepatic IP-10 expression [17, 32] with greater inflammation grade and/or fibrosis stage in liver biopsy among individuals with chronic HCV infection. In one study of individuals with chronic HCV and paired liver biopsies, plasma IP-10 levels at the time of liver biopsy were predictive of the development of fibrosis 3–5 years later [33]. However, the mechanism by which IP-10 exerts its role in the development of HCV-induced hepatic necro-inflammation is not well understood. Our findings in this current study demonstrated independent associations between higher IP-10 levels with higher ALT and HCV RNA levels in the setting of acute infection which is consistent with previous work [22, 28]. One longitudinal study describing dynamic changes of ALT and cytokines in nine individuals with acute HCV infection demonstrated a positive correlation between plasma IP-10 levels and the following week ALT levels, suggesting that an increase in IP-10 levels was associated with a subsequent increase in ALT levels [22]. In the setting of chronic HCV, similar correlations have been identified between plasma IP-10 levels with ALT [9–12] and HCV RNA levels [11, 13]. Taken together, these data suggest that the potential role of IP-10 in HCV-induced hepatic necro-inflammation might be related to both HCV cytotoxicity and liver inflammation. Further research is required to understand whether higher IP-10 levels early during acute HCV infection are predictive of subsequent fibrosis progression in chronic infection.

Our findings identified positive correlations between plasma MIP-1β levels and both ALT and HCV RNA levels in unadjusted analysis. However, in adjusted analysis higher plasma MIP-1β levels only remained associated with high HCV RNA levels (>400,000 IU/mL), suggesting that MIP-1β may be produced in response to HCV replication rather than as a result of liver inflammation, as measured by ALT elevation. This hypothesis is supported by in vitro data demonstrating that MIP-1β was induced in response to HCV RNA replication [34]. MIP-1β (CCL4) is a strong chemoattractant for T lymphocytes, monocytes, natural killer cells, and dendritic cells through interactions with CCR5, in particular and also with several other receptors including CCR1, CCR2 and CCR3 (reviewed in [8]). Increased MIP-1β levels in plasma [9, 35] and in hepatocytes [9, 32] have been demonstrated in individuals with chronic HCV infection compared to uninfected individuals. There are limited data investigating the role of MIP-1β in immuno-pathogenesis of HCV infection during acute infection. In one study of ten individuals with acute HCV infection, 70 % of participants exhibited an elevation in plasma MIP-1β levels after HCV acquisition [21]. However, another study, investigating nine individuals with acute HCV infection, showed no substantial change in plasma MIP-1β levels following infection, suggesting a limiting role for MIP-1β in lymphocyte recruitment during acute phase of HCV infection [22].

Data on the role of MIP-1β in development of necro-inflammation of the liver are also not consistent. In one longitudinal study, slow progressing liver fibrosis in HCV infection was associated with persistently lower MIP-1β plasma levels from acute to chronic infection [6]. Further, up-regulation of intra-hepatic CCR5 has been shown in fibrotic and cirrhotic livers [36, 37], while MIP-1β serum concentration was higher in individuals with chronic liver diseases compared to healthy controls and also was higher in cirrhotic individuals compared to those without cirrhosis [36]. On the other hand, in other studies, no association was found between intra-hepatic expression [32] or plasma levels [10] of MIP-1β and liver inflammation or fibrosis. One study demonstrated that while incompetent CCR5 due to a polymorphism in CCR5 gene (CCR5-∆32) was associated with mild portal inflammation, it was contradictorily associated with severe liver fibrosis [38]. This current study indicated a significant association between higher plasma MIP-1β levels and high HCV RNA levels (but not with high ALT levels) in acute infection. Similarly, although in the setting of chronic HCV, one study identified that increased plasma MIP-1 β levels were associated only with higher HCV RNA levels [9]. Taken together, it seems HCV may drive MIP-1β and any probable role of MIP-1β in liver necro-inflammation might be due to the HCV cytotoxicity, given that serum HCV RNA levels are an indirect reflection of intra-hepatic HCV replication. The mechanism linking high HCV replication to increased MIP-1β production remains to be elucidated.

This study had several limitations. First, this dataset was cross-sectional, and cytokine levels were measured at the first available HCV RNA-detectable sample, which varied in relation to the likely time since the estimated date of infection. Given this cross sectional design, it was not possible to longitudinally measure the levels of cytokines during acute HCV infection. Second, given the number of samples in this study, the cytokines and chemokines were run on two separate days and some variation was found between assay runs. However, distributions of IP-10 and MIP-1β were comparable between two assay runs and the association between ALT and HCV RNA levels and these two cytokines were unchanged after adjusting for assay run in the analyses. Third, cytokine and chemokine concentrations were measured from plasma samples, so it is possible that the levels in the blood might not reflect hepatic levels. However, a strong correlation has been demonstrated between plasma IP-10 levels and intra-hepatic expression of IP-10 mRNA in chronic HCV infection [30]. Fourth, there are many other cytokines and chemokines not assessed in this current study. As such, this study is not able to fully explain the complex signalling pathways of HCV RNA replication, liver inflammation and immuno-pathogenesis of HCV infection. Fifth, there is evidence indicating that IP-10 can undergo cleavage to form an inactive version of the protein that may act as a dominant negative by binding the CXCR3 receptor without leading to chemotaxis and can act as an antagonist [39, 40]. Measurement of the cleaved and uncleaved fractions of IP-10 requires storage of plasma in specialized tubes to avoid post-collection cleavage. Unfortunately, the samples used in this study were not stored to allow for measurement of cleaved IP-10, so this could not be evaluated. Sixth, it is possible that long-term storage may have led to reduced levels of some cytokines and chemokines [41]. However, participant samples were stored identically with a minimal number of freeze-thaws, so it is not anticipated that this would have had an effect on the observed results between the association of ALT and HCV RNA levels with IP-10 and MIP-1β levels. Further, data on liver fibrosis levels were not available in our participants. As such, we were not able to assess the association of IP-10 and MIP-1β levels with liver fibrosis in this study. Lastly, given that 38 % of our participants were co-infected with HIV, we used two approaches to control for the potential effect of HIV on ALT and HCV RNA levels with IP-10 and MIP-1β levels, including stratification and adjustment in multivariate analysis. However, in both analyses, IP-10 and MIP-1β levels were associated with ALT and HCV RNA levels, regardless of HIV status.

## Conclusion

In conclusion, this study demonstrated that during recent HCV infection, higher ALT levels were associated with increased IP-10 levels, while high HCV RNA levels were associated with increased plasma IP-10 and MIP-1β levels. These data improve our understanding of immuno-pathogenesis of HCV infection, suggesting that the potential roles of IP-10 and MIP-1β in HCV immuno-pathogenesis start from acute phase of infection, possibly in relation with liver inflammation (IP-10) and HCV cytotoxicity (IP-10 and MIP-1β). These hypotheses need to be investigated through further research to elucidate the mechanism linking IP-10 and MIP-1β with HCV replication and liver inflammation. Further research is also needed to investigate whether higher IP-10 and MIP-1β levels during acute HCV infection are predictive of subsequent fibrosis progression in chronic infection.

## Methods

### Study participants

The Australian Trial in Acute Hepatitis C (ATAHC) was a prospective study of the natural history and treatment of recently acquired HCV infection. The ATAHC study design has been described previously in detail [42]. Recent HCV infection (i.e., acute or early chronic infection) was defined by an initial positive anti-HCV antibody test within 6 months of enrolment and either 1) a negative anti-HCV antibody test within two years prior to the initial positive anti-HCV antibody test or 2) acute clinical hepatitis within 12 months before the initial positive anti-HCV antibody result. Acute clinical infection was defined by symptomatic seroconversion illness or peak ALT levels greater than 400 IU/L at or before the time of HCV diagnosis.

In the current study, ATAHC participants with available plasma samples and HCV RNA detected at the time of HCV detection were included. Cytokines and chemokines were measured in plasma samples at the time of HCV detection using a multiplex assay (see below). The ATAHC protocol was reviewed and approved by Human Research Ethics Committees of St. Vincent’s Hospital, Sydney and the University Health Network, and all participants provided informed written consent. ATAHC was performed according to the World Medical Association Declaration of Helsinki. The study was registered with clinicaltrials.gov registry (NCT00192569).

### Measurement of plasma cytokines and chemokines

Three human cytokine multiplex bead array assay kits utilizing technology licensed by Luminex were purchased from Bio-Rad (Gladesville, Australia) to measure the following cytokines and chemokines:

- *Measured with Bio*-*Plex human TH17 15*-*plex*: interleukin-1 beta (IL-1β), IL-4, IL-6, IL-10, IL-17A, IL-17 F, IL-21, IL-22, IL-23, IL-25,IL-31, IL-33, interferon-gamma (IFN-γ), soluble CD40 ligand (sCD40L), and tumor necrosis factor alpha (TNF-α)
- *Measured with Bio*-*Plex human cytokine Group I 9*-*plex*: IL-2, IL-8, eotaxin-1 (or CCL11), IFN-γ, interferon-gamma inducible protein-10 (IP-10, or CXCL10), monocyte chemotactic protein 1 (MCP-1, or CCL2), macrophage inflammatory protein 1 alpha (MIP-1α, or CCL3), MIP-1β (or CCL4), and regulated upon activation normal T-cell expressed and presumably secreted (RANTES or CCL5)
- *Measured with Bio*-*Plex human cytokine Group II 3*-*plex*: IL-18, TNF-β, and TNF-related apoptosis-inducing ligand (TRAIL, or TNFSF10).

The protocol was performed as per the manufacturer’s instructions as previously described [43, 44], with samples centrifuged at 10,000 × g for 10 minutes at 4 °C to remove platelets and precipitates, after which the supernatants were diluted four times with assay diluents. The assay was performed in a 96-well filter plate, using all the assay components provided. All incubation steps were performed at room temperature and in the dark to protect the beads from light and washes were performed using a vacuum manifold. For the detection of cytokines and chemokines, the samples were then incubated for 10 minutes while shaken at 850 rpm with streptavidin conjugated to the fluorescent protein, Streptavidin-PE. After subsequent washing in order to remove the unbound Streptavidin-PE, the beads (minimum of 50 beads per cytokine) were analysed on the Bio-Plex 200 instrument, which monitored the spectral properties of the beads while simultaneously measuring the amount of fluorescence associated with R-phycoerythrin. The raw data was analysed using the Bio-Plex Manager software, v6.1 (Bio-Rad) [21]. Cytokine standards supplied by the manufacturer were used to calculate the concentrations of the samples. The lower limits of quantification for all cytokines and chemokines are summarized in Additional file 1: Table S1. Due to the large number of samples, they were split randomly in two sets and processed on two 96 well plates (assay runs). Each day, one set was run for all three cytokines and chemokines panels. Lot numbers for multiplex bead array assay kits and procedure used were identical between assay runs.

### HCV RNA and genotype testing

The presence of HCV RNA was assessed with a qualitative HCV RNA assay (TMA assay; Versant, Bayer, Australia; lower limit of detection, 10 IU/mL) and if positive HCV RNA levels were assesses with a quantitative HCV RNA assay (Versant HCV RNA 3.0; Bayer, Australia; lower limit of detection, 615 IU/mL). HCV genotype (Versant LiPa; Bayer, Australia) was assessed for participants with detectable HCV RNA.

### Statistical analysis

Log_10_ transformed values of ALT levels (log_10_ IU/L) and cytokines levels (log_10_ pg/mL) were used in analysis given the distribution of the actual values were not normal. Given that HCV RNA levels (IU/mL) and log_10_ transformation of HCV RNA levels (log_10_ IU/mL) were not normally distributed, non-parametric statistical methods were the basis of analysis of HCV RNA levels.

The correlation of ALT with plasma cytokine levels were assessed using Pearson correlation, while the correlation of HCV RNA with plasma cytokine levels were assessed using Spearman’s rank-order correlation. If a plasma cytokine level was below the lower level of quantification, the midpoint between zero and the lowest level of quantification was imputed (Additional file 1: Table S1). Plasma levels of four cytokines (IL-23, IFN-γ, TNF-β, and MIP-1a) were below the level of quantification in ≥20 % of individuals, for each of those the distribution of ALT and HCV RNA levels were compared between individuals with quantifiable and those with unquantifiable cytokine levels (Wilcoxon-Mann–Whitney test).

Overall, the association of ALT and HCV RNA levels with 27 plasma cytokines levels were assessed. Whether adjustments are needed for multiple comparisons is a matter of controversy given that multiple comparison testing might inflate type 1 error while adjustments for multiple comparisons inflate type 2 error [45–48]. To account for multiple comparisons, a moderately conservative significance level (*alpha* = 0.01) was used. In a sensitivity analysis, using Bonferroni correction for multiple comparisons, an *alpha* = 0.05/27 = 0.002 was also used, but the findings were similar.

Cytokines demonstrating significant correlations with ALT and/or HCV RNA levels were included in linear regression analysis. Linear regression models were fit to assess the correlation of ALT levels (log IU/L) and HCV RNA levels (categorised by tertile: <8,500 IU/mL, 8,500-400,000 IU/mL, >400,000 IU/mL) with each plasma cytokine levels (log pg/mL). In the adjusted models, the correlation of ALT and HCV RNA levels with cytokine levels was adjusted for potential confounders including sex, age, estimated duration of infection at acute HCV detection, symptomatic illness at acute HCV detection, HIV co-infection, *interferon*-*lambda rs12979860* genotype, HCV genotype, and assay run. Potential confounders were adjusted for, including variables associated with either of ALT, HCV RNA, or cytokine levels in this study (*P* < 0.200). To account for potential unmeasured confounders introduced by different assay runs and sample set, the models were also adjusted for assay run. All analyses were performed using Stata v12.0 (College Station, TX, United States).

## Additional file

Additional file 1: **Supplementary Material**. (DOCX 38 kb)

## Abbreviations

ALT: alanine aminotransferase
ATAHC: Australian trial in acute hepatitis C
HCV: hepatitis C virus
IFN: interferon-gamma
IL: interleukin
IP-10: interferon-gamma inducible protein-10
IQR: inter-quartile range
MCP: monocyte chemotactic protein
MIP: macrophage inflammatory protein
RANTES: regulated upon activation normal T-cell expressed and presumably secreted
sCD40L: soluble CD40 ligand
TNF: tumor necrosis factor
TRAIL: TNF-related apoptosis-inducing ligand
