# Supplementary material for: Alanine aminotransferase, HCV RNA levels and pro-inflammatory and pro-fibrogenic cytokines/chemokines during acute hepatitis C virus infection
Source: Virol J. 2016 Feb 24;13:32. doi: 10.1186/s12985-016-0482-x (PMC4765111; doi:10.1186/s12985-016-0482-x)
Supplement: Supplementary file 1 — Supplementary Material. (DOCX 38 kb) [file 12985_2016_482_MOESM1_ESM.docx]

**SUPPLEMENTARY MATERIAL**

**Supplementary Table 1. The lowest level of detection for measurement of plasma cytokine and chemokine levels**

| Cytokine | Lowest level of detection (pg/mL) |
| --- | --- |
| IL-1β | 0.274 |
| IL-2 | 0.479 |
| IL-4 | 5.113 |
| IL-6 | 1.173 |
| IL-8 | 1.346 |
| IL-10 | 2.108 |
| IL-17A | 1.681 |
| IL-17F | 9.629 |
| IL-18 | 0.741 |
| IL-21 | 15.798 |
| IL-22 | 4.682 |
| IL-23 | 29.334 |
| IL-25 | 1.233 |
| IL-31 | 12.631 |
| IL-33 | 3.387 |
| IFN-γ | 46.458 |
| IFN-γ2 | 41.454 |
| TNF-α | 0.283 |
| TNF-β | 1.154 |
| TRAIL | 2.570 |
| sCD40L | 2.551 |
| CXCL10 (IP-10) | 2.376 |
| CCL2 (MCP-1) | 1.037 |
| CCL3 (MIP-1α) | 1.151 |
| CCL4 (MIP-1β) | 1.326 |
| CCL5 (RANTES) | 5.151 |
| CCL11 (eotaxin) | 1.774 |

**Supplementary Table 2: Factors associated with ALT levels (log IU/L) in ATAHC participants with detectable HCV RNA at the time of acute HCV detection (n=117)**

|  | Estimated Mean difference (95% CI) | *P* |
| --- | --- | --- |
| **Sex** |  |  |
| Male | Reference |  |
| Female | -0.52 (-0.71, -0.33) | <0.001 |
| **Age^*^** |  |  |
| <30 years | Reference |  |
| 30-39 years | 0.13 (-0.09, 0.35) | 0.237 |
| ≥40 years | 0.23 (0.01, 0.44) | 0.043 |
| **Symptomatic acute HCV** |  |  |
| No | Reference |  |
| Yes | 0.18 (0.00, 0.368) | 0.048 |
| **Estimated duration of infection** |  |  |
| <26 weeks | Reference |  |
| ≥26 weeks | -0.31 (-0.49, -0.13) | 0.001 |
| **HIV co-infection** |  |  |
| Negative | Reference |  |
| Positive | 0.26 (0.08, 0.45) | 0.006 |
| ***Interferon lambda rs12979860* genotype** |  |  |
| TT/CT | Reference |  |
| CC | 0.15 (-0.03, 0.34) | 0.098 |
| **HCV RNA level^**^** |  |  |
| < 8,500 IU/mL | Reference |  |
| 8,500 - 400,000 IU/mL | 0.44 (0.25, 0.63) | <0.001 |
| > 400,000 IU/mL | 0.63 (0.44, 0.83) | <0.001 |
| **HCV genotype^†^** |  |  |
| Genotype 1 | Reference |  |
| Genotype 2 | 0.09 (-0.32, 0.50) | 0.655 |
| Genotype 3 | 0.10 (-0.09, 0.29) | 0.303 |
| Other | -0.41 (-1.10, 0.28) | 0.240 |

*Overall *P*=0.119

**Overall *P*<0.001

†Overall *P*=0.0.420

**Supplementary Table 3: Factors associated with HCV RNA levels (IU/mL) in ATAHC participants with detectable HCV RNA at the time of acute HCV detection (n=117)**

|  | **HCV RNA levels (IU/mL)** | | | *P*^**^ | *P*^†^ |
| --- | --- | --- | --- | --- | --- |
|  | <8,500 (n=39) n (%)*^*^* | 8,500 - 400,000 (n=39) n (%)*^*^* | >400,000 (n=39) n (%)*^*^* |  |  |
| **Sex** |  |  |  | 0.031 | 0.034 |
| Male | 24 (61) | 30 (77) | 34 (87) |  |  |
| Female | 15 (38) | 9 (23) | 5 (13) |  |  |
| **Age** |  |  |  | 0.031 | 0.007 |
| <30 years | 17 (44) | 20 (51) | 11 (28) |  |  |
| 30-39 years | 14 (36) | 11 (28) | 9 (23) |  |  |
| ≥40 years | 8 (20) | 8 (20) | 19 (49) |  |  |
| **Symptomatic acute HCV** |  |  |  | 0.634 | 0.791 |
| No | 21 (54) | 25 (64) | 22 (56) |  |  |
| Yes | 18 (46) | 14 (36) | 17 (44) |  |  |
| **Estimated duration of infection** |  |  |  | 0.252 | 0.69 |
| <26 weeks | 26 (67) | 19 (49) | 24 (61) |  |  |
| ≥26 weeks | 13 (33) | 20 (51) | 15 (38) |  |  |
| **HIV co-infection** |  |  |  | 0.063 | 0.031 |
| Negative | 25 (64) | 29 (74) | 19 (49) |  |  |
| Positive | 14 (36) | 10 (26) | 20 (51) |  |  |
| ***Interferon lambda rs12979860* genotype** |  |  |  | 0.282 | 0.433 |
| TT/CT | 17 (44) | 22 (59) | 17 (44) |  |  |
| CC | 22 (56) | 15 (40) | 22 (56) |  |  |
| **Mean ALT level (SD), log IU/L** | 1.9 (0.4) | 2.3 (0.4) | 2.5 (0.4) | <0.001 | <0.001 |
| **HCV genotype** |  |  |  | 0.933 | 0.714 |
| Genotype 1 | 22 (59) | 22 (56) | 23 (59) |  |  |
| Genotype 2 | 2 (5) | 1 (3) | 3 (8) |  |  |
| Genotype 3 | 12 (32) | 15 (38) | 13 (33) |  |  |
| Other | 1 (3) | 1 (3) | 0 (0) |  |  |

*Percentages indicate column percentages

**For comparison across three groups;

†For comparing HCV RNA levels >400,000 IU/ml vs. ≤400,000 IU/mL

**Supplementary Table 4: Correlation of plasma IP-10 and MIP-1β levels (log pg/mL) with ALT and HCV RNA levels, stratified by HIV co-infection status**

|  | **HCV mono-infection**  **(n=73)** | | **HCV/HIV co-infection**  **(n=44)** | |
| --- | --- | --- | --- | --- |
|  | **Estimated Mean difference^*^ (95% CI)** | ***P*** | **Estimated Mean difference^*^ (95% CI)** | ***P*** |
| **IP-10 level, log pg/mL** | | | | |
| **ALT level, log IU/L** | 0.17 (0.06, 0.28) | 0.003 | 0.26 (0.10, 0.41) | 0.002 |
| **HCV RNA level** |  |  |  |  |
| < 8,500 IU/mL | Reference |  | Reference |  |
| 8,500 - 400,000 IU/mL | 0.03 (-0.08, 0.15) | 0.549 | 0.06 (-0.15, 0.27) | 0.584 |
| > 400,000 IU/mL | 0.25 (0.12, 0.38) | <0.001 | 0.27 (0.09, 0.45) | 0.004 |
| **MIP-1β level, log pg/mL** | | | | |
| **ALT level, log IU/L** | 0.17 (0.06, 0.29) | 0.003 | 0.12 (-0.8, 0.31) | 0.249 |
| **HCV RNA level** |  |  |  |  |
| < 8,500 IU/mL | Reference |  | Reference |  |
| 8,500 - 400,000 IU/mL | 0.17 (0.04, 0.30) | 0.010 | 0.07 (-0.17, 0.31) | 0.581 |
| > 400,000 IU/mL | 0.17 (0.03, 0.31) | 0.020 | 0.32 (0.11, 0.52) | 0.004 |

*β coefficient
